# *DEPDC1* as a crucial factor in the progression of human osteosarcoma

**DOI:** 10.1002/cam4.5340

**Published:** 2022-12-07

**Authors:** Lin Shen, Han Li, Ronghan Liu, Chendan Zhou, Morgan Bretches, Xuan Gong, Laitong Lu, Ying Zhang, Kai Zhao, Bin Ning, Shang‐You Yang, Aijun Zhang

**Affiliations:** ^1^ Department of Orthopaedics Central Hospital Affiliated to Shandong First Medical University Jinan Shandong China; ^2^ Department of Endocrinology The First Affiliated Hospital of Xiamen University Xiamen Fujian China; ^3^ Department of Pediatrics Qilu Hospital, Shandong University Jinan China; ^4^ Department of Orthopaedic Surgery University of Kansas School of Medicine‐Wichita Wichita Kansas USA; ^5^ Department of Biological Sciences Wichita State University Wichita Kansas USA; ^6^ University of Texas Southwestern Medical Center Dallas Texas USA

**Keywords:** bioinformatics, *DEPDC1*, mouse model, osteosarcoma, poor prognosis

## Abstract

**Objective:**

Novel therapeutic strategies are emerging with the increased understanding of the underlying mechanisms of human osteosarcoma. This current study tends to decipher the potentially critical role of DEP domain‐containing 1 (DEPDC1), a tumor‐related gene, during the progression of osteosarcoma.

**Methods:**

Bioinformatics analysis of 25,035 genes from the National Center for Biotechnology Information (NCBI) databases was performed to screen differentially expressed genes between osteosarcoma and normal control groups, complemented by the examination of 85 clinical osteosarcoma specimens. Furthermore, the manipulation of *DEPDC1* expression levels by using silencing RNA (siRNA) or lentiviral vector intervention on human osteosarcoma cells was performed to reveal its role and interactions in in vitro and in vivo settings.

**Results:**

Gene expression profile analysis and immunohistochemical (IHC) examination suggested that *DEPDC1* is highly expressed in human osteosarcoma cells and tumor tissue. The silencing of *DEPDC1* arrested osteosarcoma cell proliferation, promoted apoptosis, and ceased tumor metastasis. Studies involving clinical human osteosarcoma cases exhibited a strong correlation of *DEPDC1* over‐expressed osteosarcoma specimens with a reduced patient survival rate.

**Conclusions:**

Collectively, this study demonstrated that *DEPDC1* is a critical driver in the promotion of osteosarcoma progression and results in poor patient prognosis. Genetically targeting or pharmacologically inhibiting *DEPDC1* may serve as a promising strategy for treating human osteosarcoma.

## Graphical abstract

In this study, we determined that DEPDC1 could promote the proliferation and migration of osteosarcoma cells in vivo and in vitro. Besides, DEPDC1 can accurately predict the clinical features and prognosis of patients, which may provide a new target for the diagnosis and treatment of human osteosarcoma.

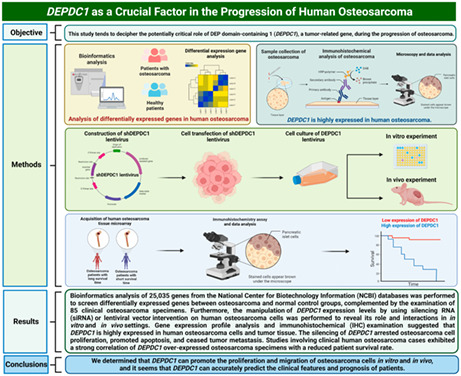

## 1 INTRODUCTION

Osteosarcoma is a common, primarily malignant bone tumor in children and adolescents. Wide surgical resection in addition to adjuvant radiotherapy and chemotherapy are the main regimes for osteosarcoma treatment. However, the side effects of chemotherapy and the drug resistance of osteosarcoma often lead to poor prognosis.^1^, ^2^, ^3^ Thus, deciphering the molecular mechanisms underlying the progression of osteosarcoma is urgently needed.

Recent studies have focused on exploring the critical oncogenes that promote the occurrence and development of osteosarcoma. The occurrence and development of a malignant tumor is a continuous and complex process, with a variety of gene expression changes being noted. Many studies have found that *XIAP, COX‐2, Livin*, and many other genes are highly expressed in osteosarcoma, while expressions of *Caspase‐3* and *TP53* genes are decreased.^4^, ^5^, ^6^, ^7^, ^8^ *XIAP* and *Livin* are associated with the apoptosis inhibitory protein family and directly affect Caspase‐3, Caspase‐7i, and Caspase‐9 to initiate the inhibition of tumor cell apoptosis.^9^, ^10^, ^11^, ^12^ High expression of *COX‐2* is mainly involved in promoting tumor angiogenesis and inhibiting tumor apoptosis.^13^ As a tumor suppressor gene, *TP53* induces the apoptosis of tumor cells through the cytoplasmic proteins Bax/Bcl2, Fas/Apol, and IGF‐BP3.^14^ According to the change in spatial conformation, mutant *TP53* loses the regulatory effect on cell growth, apoptosis, and DNA repair, and transforms itself from a tumor suppressor gene to an oncogene.^10^ It has been confirmed that the gene regulation network of osteosarcoma mainly suppresses the apoptosis of tumor cells, but the underlying mechanism in proliferation and metastasis of osteosarcoma is still unclear. The objectives of this current study are to analyze the differentially expressed genes that interact in promoting osteosarcoma cell proliferation and metastasis using strategies that include the bioinformatical analysis of National Center for Biotechnology Information (NCBI) GenBank data, assessment of clinical osteosarcoma specimens, and an experimental osteosarcoma animal model, and in vitro mechanistic experimentation. Interestingly, a few genes have been identified to be interactively associated with the progression of osteosarcoma.

DEP domain‐containing 1 (*DEPDC1*) is a newly identified tumor‐related gene. Recent studies have shown that *DEPDC1* is overexpressed in many types of malignant tumors such as bladder cancer, breast cancer, lung adenocarcinoma, and colorectal cancer.^9^, ^11^, ^13^, ^14^, ^15^ One important report illustrated that a complex formed by *DEPDC1* and zinc finger protein 224 (ZNF224) can diminish the expression of zinc finger protein A20 to weaken the inhibitory effect of A20 on the degradation of the inhibitor of NF‐κB (I‐κB), thus promoting the translocation of NF‐κB into the nucleus to activate the expression of related genes, ultimately playing an anti‐apoptotic physiological role in bladder cancer.^9^ Oral squamous cell carcinoma (OSCC) is a prevalent malignancy, and a recent study observed that *DEPDC1* protein was overexpressed in OSCC tissue with a demonstrated correlation between smoking status and upregulated *DEPDC1* expressions.^16^ However, there is no published evidence as to whether *DEPDC1* plays a role in the development and progression of human osteosarcoma. Preliminary data analysis of the online database in our laboratory has suggested that *DEPDC1* may also be overexpressed in human osteosarcoma tissue. It is worthwhile to understand its role and molecular pathway during the progression of osteosarcoma. We hypothesize that *DEPDC1* is crucial in the progression and prognosis of osteosarcoma, while the silencing of *DEPDC1* expression can halt osteosarcoma cell proliferation and tumor progression.

## 2 MATERIALS AND METHODS

### 2.1 Bioinformatics analysis of differential gene expressions in human osteosarcoma

Eight datasets (species: *Homo sapiens*) were downloaded from the NCBI Gene Expression Omnibus (GEO) database: GSE11414, GSE12865, GSE14359, GSE16088, GSE19276, GSE28424, GSE36001, and GSE9508 (data download time: January, 2019). The Limma software package (v. 3.10.3) was used to analyze the differences in gene expression between the osteosarcoma group and the normal group to obtain the corresponding *p*‐ and logFC values. The Benjamini‐Hochberg method^17^ was performed for multiple tests and corrections, and the corrected *p*‐value (namely the adjusted *p*‐value) was obtained.

### 2.2 Clinical samples and survival time analysis

A total of 85 osteosarcoma tissue specimens including their adjacent normal tissues were collected along with de‐identifiable clinical chart information. Among them, 75 specimens were from Jinan Central Hospital, and 10 were from the Xi'an Best Biotechnology Co., Ltd. This multi‐institutional collaborative human investigation project was approved by ethics committees at the individual institutions. The receiver operating characteristic (ROC) curve was used to analyze the relationship between protein expression and survival time. The values of ROC curves with the maximized sum of sensitivity and specificity were used as thresholds to separate the high and low expressions of the *DEPDC1* gene.

### 2.3 Immunohistochemical staining and scoring

Immunohistochemical (IHC) staining and analysis of the clinical specimens were performed with antibodies against human *DEPDC1* (1:100; Abcam). QuantCenter software (v 2.0, Hungary) and Pannoramic Viewer software (v 1.15.4, Hungary) were used to quantitatively score tissue slices. We adopted the histochemistry score (H‐score), a histological scoring method to quantitate the IHC results.^18^, ^19^ The number of positive cells and their staining intensity in each section were converted to corresponding values to achieve the purpose of semi‐quantitative staining. The formula to calculate the staining intensity is as follows: H‐score = (percentage of cells of weak intensity×1) + (percentage of cells of moderate intensity×2) + (percentage of cells of strong intensity×3). The maximum H‐score value is 300, and the minimum value is 0. H‐score values of 150 or more are considered the high expression, whereas H‐score values less than <150 are considered the low expression.

### 2.4 Cell culture and reagents

Human osteosarcoma cell lines HOS(#20210713–17), MG‐63 (#20210531–09), and Saos‐2(#20210810–21) were commercially obtained from Genechem Co., Ltd. HOS cells were initially isolated and established from the osteosarcoma tissue of a 13‐year‐old white woman. MG‐63 cells are derived from a 14‐year‐old white male with osteosarcoma. Saos‐2 cell is one of many human tumor cell lines isolated and identified by Fogh J and Trempe G. The cell comes from osteosarcoma tissue of an 11‐year‐old white woman. The human osteoblast cell line hFOB1.19 (#20170410–07) was purchased from Zhongqiao Xinzhou Biotechnology Co., Ltd. and immortalized by SV40 large T antigen transformation. All cell lines were authenticated by the vendors using short tandem repeat (STR) DNA profiling analysis prior to shipping (Appendix S1). Cells were cultured in Dulbecco's modified Eagle's medium supplemented with 10% fetal bovine serum, streptomycin (100 mg/ml), and penicillin (100 U/ml), and maintained at 37°C in a 5% CO_2_ atmosphere.

### 2.5 Lentiviral and plasmid vectors for in vitro *DEPDC1* alteration

*DEPDC1* siRNA plasmids (psc33550, Genechem), as well as *DEPDC1* short hairpin RNA (shRNA, GSGC0121294, Genechem) lentiviral particles (sh*DEPDC1*), were designed and constructed by the Genechem Co., Ltd. for *DEPDC1* silencing studies. The sh*DEPDC1* and shCtrl viral or plasmid vectors were co‐cultured with the osteosarcoma cells at a multiplicity of infection (MOI) of 25 and 20, respectively.

### 2.6 MTT, EdU staining, and clonogenic survival assays

Cells infected with either shCtrl or *DEPDC1*‐siRNA lentivirus were cultured at 5 × 10^4^/well in a 96‐well plate at 37°C in a 5% CO_2_ atmosphere for 5 days before being assayed for 3‐(4,5‐dimethylthiazol‐2‐yl)‐2,5‐diphenyltetrazolium bromide (MTT) cell proliferation, as reported previously.^20^ To determine newly synthesized DNA, the 5‐ethynyl‐2′‐deoxyuridine (EdU) staining assay was performed with a BeyoClick™ EdU cell Proliferation Kit with Alexa Fluor 594 (Beyotime, Cat. No.: C00788L) as detailed elsewhere.^21^ Briefly, 10 μM of EdU was applied to the cultures for 2 h at 37°C and 5% CO_2_ before the cells were fixed in 4% paraformaldehyde and co‐stained with DAPI (4′,6‐diamidino‐2‐phenylindole) for nuclear visualization. For the clonogenic assay, 1 × 10^3^ HOS and MG‐63 cells infected with sh*DEPDC1* or shCtrl lentivirus were spread into each well of six‐well culture plates. The cells were incubated at 37°C and 5% CO_2_ for 14 days. The cell colonies were fixed with 4% paraformaldehyde and stained with Giemsa (MilliporeSigma). Colonies consisting of >50 cells were counted.

### 2.7 Apoptosis analysis

HOS or MG‐63 cells were seeded into six‐well plates at a density of 1.5 × 10^5^cells/well, incubated for 24 h, and grouped as shCtrl and sh*DEPDC1*. After 24 h, the cells from all groups were harvested and washed twice with cold phosphate‐buffered saline (PBS). Apoptotic cells were distinguished by Annexin V‐7AAD/PI dual staining and an apoptosis detection kit from Keygen Biotech. Flow cytometry was also performed to detect apoptotic cells (FCM, BD FACS Calibur™; BD Biosciences). All experiments were performed in triplicate.

### 2.8 Cell culture scratch assay

A total of 1.5 × 10^6^ HOS or MG‐63 cells in the log phase were seeded into each well of a six‐well plate as shCtrl and sh*DEPDC1* groups, respectively. When the cell density reached approximately 80%, cells from all four groups were rinsed twice with PBS before scratching a line in the center of each well with a pipette tip. Each well was imaged using ImageJ software (NIH), and the total initial wounded area (scratched line) at 0 h (S0h) was calculated. Cells were left to continue culturing in the serum‐free medium for 48 h. The culture wells were imaged, and the total final wounded areas at 48 h (S48h) were recorded.

### 2.9 Transwell assay

HOS or MG‐63 cells invasion activities were determined using a 24‐well Transwell chamber (cat.no.3422; Corning Costar Corp.) with Matrigel (cat.no.082704; ABW). A total of 1 × 10^5^ HOS or MG‐63 cells prepared in 400 μl serum‐free media were seeded into the upper chamber of the Transwell insert. A total of 600ul Transwell chamber with Matrigel (TCM) was added to the lower chamber of the Transwell insert. After incubation for 48 h, the cells remaining in the upper chamber of the Transwell insert were removed. Cells on the underside of the chamber were fixed with 4% paraformaldehyde at room temperature for 30 min and stained with 0.1% crystal violet at room temperature for 15 min. Invaded cells were subsequently counted under an inverted fluorescence microscope(Nikon TE2000; Nikon Corp.) (magnification, ×200) in six different fields of view for each sample.

### 2.10 Quantitative real‐time PCR and western blotting

Messenger RNA (mRNA) expression levels of the target genes were determined by the quantitative real‐time polymerase chain reaction (qRT‐PCR) using SYBR Green PCR Master Mix, as described previously.^22^ The results were normalized to human GAPDH (glyceraldehyde 3‐phosphate dehydrogenase) mRNA expression. Three independent experiments were performed for each test. The protein expression level of specimens was examined using western blotting, as detailed previously.^23^ After the SDS‐PAGE (sodium dodecyl sulfate‐polyacrylamide gel electrophoresis) and protein transfer onto polyvinylidene difluoride membranes, primary anti‐*DEPDC1* (ab183620, 1:500, polyclonal; Abcam) was applied overnight at 4°C. GAPDH (sc‐47,724, 1:1000, polyclonal; Santa Cruz Biotechnology) or β‐actin (sc‐8432, 1:1000, polyclonal; Santa Cruz Biotechnology) antibodies were used as controls.

### 2.11 Experimental osteosarcoma mouse model

All animal experimentation protocols were approved by the Institutional Ethics Committee.

There were 24 Balb/c nude mice (Beijing Vital River Laboratory Animal Technology Co. Ltd.) all of which were similar in age and weight. The selected nude mice were randomly divided into the NC group and sh*DEPDC1* group (12 female mice per group). For xenograft models, MG‐63 cells were used. The cells were trypsinized and resuspended in PBS (density equal to 5 × 10^7^ cells/ml). The partial skin of nude mice was sterilized and then injected with 0.1 ml cell suspension at the right sub‐axillary region in each nude mouse. Mice were sacrificed using the standard cervical dislocation method, and subsequent experiments were performed after repeated confirmation that the mice were not breathing, feeling, or conscious and that the mice were dead to avoid suffering. Body weight and tumor diameter were measured every other day. Tumor size was measured daily to calculate the volume based on the formula (length [L] × width [W]^2^)/2. On day 37, the tumors were harvested, weighed, and fixed in 10% formalin before being processed for paraffin‐embedded tissue sectioning. The 5‐μm‐thick sections were stained for hematoxylin and eosin (H&E) and IHC analysis. No tumor volume was >2000 mm^3^.

### 2.12 Statistical analysis

Data were processed in GraphPad Prism 6.0 (GraphPad Software) and SPSS v. 20.0 (IBM Corp.). The Kaplan–Meier method was used for survival analysis. For comparing the two groups, the student's *t*‐test was applied if no significantly different variances were present; otherwise, the Mann–Whitney test was used. One‐way ANOVA with Bonferroni's test was used to compare >2 group means. Data are expressed as means ± standard deviation (SD). Differences were considered statistically significant at *p* < 0.05.

## 3 RESULTS

### 3.1 Highly expressed *DEPDC1* in human osteosarcoma tissues and cells

**FIGURE 1:**
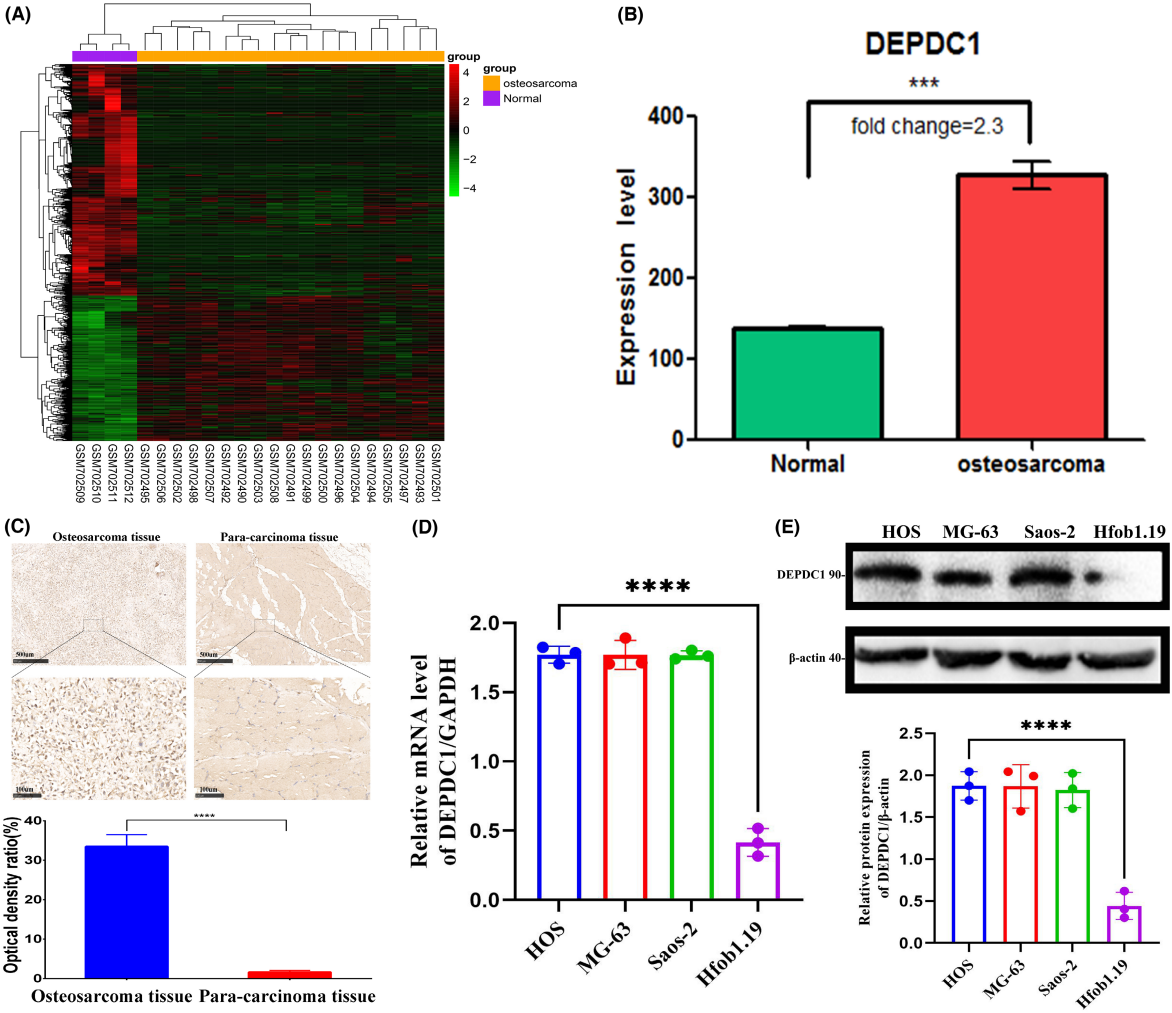
*DEPDC1* highly expressed in GEO database bioinformatics analysis, and clinical osteosarcoma specimens and osteosarcoma cell lines. (A) Heat map across the human osteosarcoma among GEO database. (B) Bar chart showing expression levels of *DEPDC1*. (C) Representative immunohistochemical (IHC) staining (scale bar 100 μm) against *DEPDC1* protein on osteosarcoma patient tumor biopsy sections (left panel) versus adjacent normal tissues (right panel), with quantification bar chart at the bottom. (D) The plot of real‐time PCR analysis of *DEPDC1* expression on osteosarcoma cells (HOS, MG‐63, and Saos‐2) and normal osteoblast controls (hFOB1.19). (E) Representative western blot images of cell lysates with antibodies against *DEPDC1* on the top row, with the lower panel indicating the normalized quantification (***p < 0.01, ****p < 0.001).

To explore the crucial oncogenes related to osteosarcoma progression, we adopted bioinformatics techniques to analyze the differential gene expressions between an osteosarcoma group and a normal group from the GEO database including 25,035 annotated genes. A total of 1355 differentially expressed genes were screened out (523 upregulated genes and 832 downregulated genes) (Figure 1A). For the combined adjusted *p*‐ and logFC values, the logFC value of *DEPDC1* was the largest (logFC = 1.204), with a corresponding adjusted *p*‐value of <0.05. It is shown that the relative expression level of *DEPDC1* in the osteosarcoma group was 2.3 times higher than that in the control group (Figure 1B). IHC examination on the clinical osteosarcoma biopsy specimens revealed ubiquitous *DEPDC1*+ staining compared to the adjacent normal tissue (Figure 1C). Real‐time PCR and western blot assessments on osteosarcoma cell lines (HOS, MG‐63, and Saos‐2) also indicated significant overexpression of *DEPDC1* at mRNA and protein levels, in comparison with the human osteoblastic cell line Hfob1.19 (Figure 1D,E).

### 3.2 Suppression of *DEPDC1* expression halting osteosarcoma cell proliferation and migration, and promoting apoptosis

**FIGURE 2:**
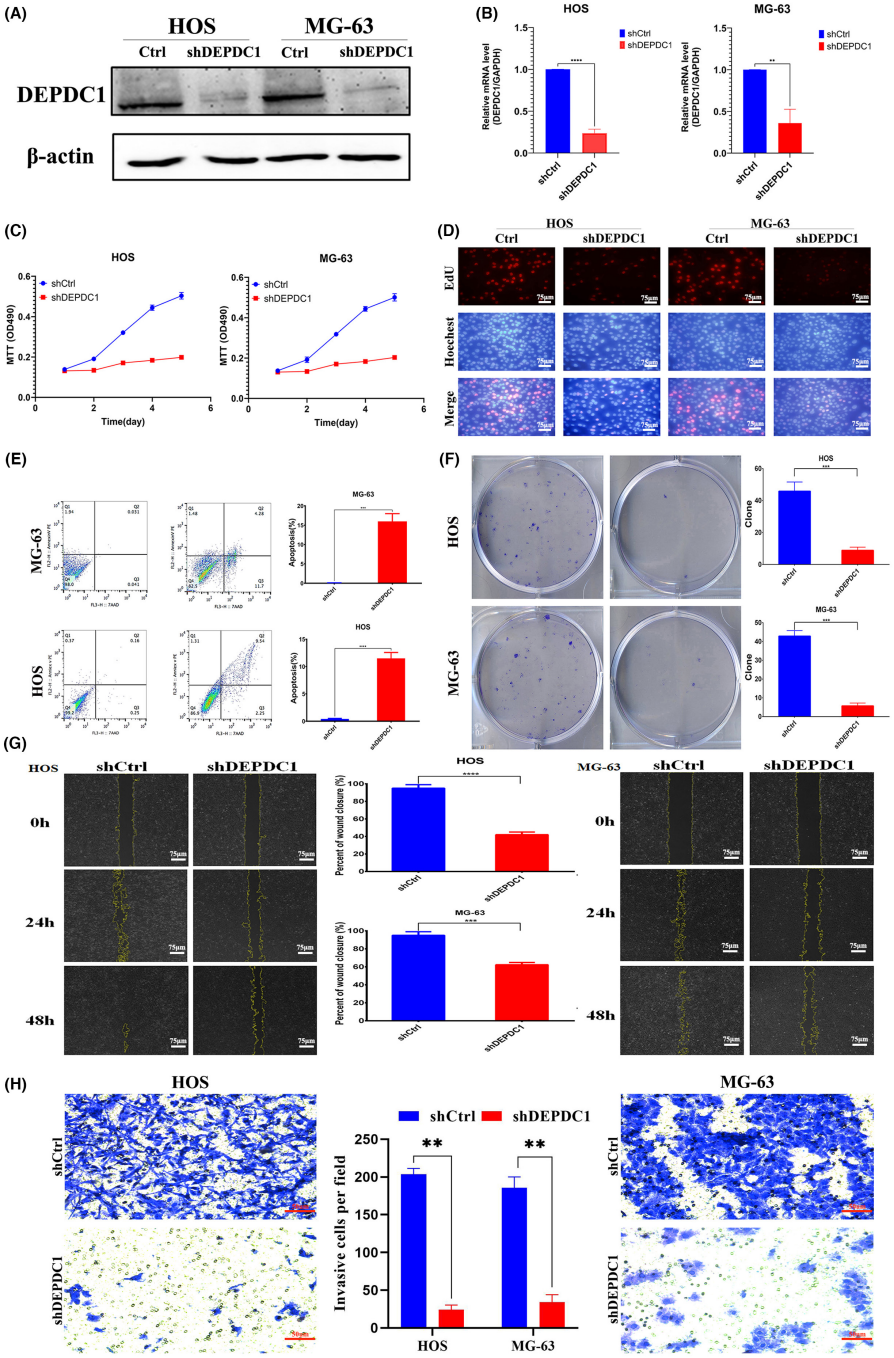
Downregulation of *DEPDC1* inhibiting osteosarcoma cell proliferation and migration, and promoting apoptosis. (A) Western blotting and (B) real‐time PCR showing down‐regulation of *DEPDC1* in sh*DEPDC1* groups. (C) MTT assay showing diminishment of cell proliferations after knockdown of *DEPDC1* expression in HOS and MG‐63 cells. (D) EdU assay showing knockdown of *DEPDC1* expression could lead to cell cycle arrest in osteosarcoma cell lines. (E) Flow cytometry quantification of apoptotic cell percentages among groups. (F) Cell colony visualization and quantification of HOS and MG‐63 cells with control or sh*DEPDC1* interventions. Cell scratch assay **(**G) and Transwell assay (H) to evaluate cell migration and cell invasion capacities of HOS and MG‐63 osteosarcoma cells after silencing of *DEPDC1* expression. (***p < 0.01, ****p < 0.001).

Lentiviral vectors‐mediated silencing of *DEPDC1* expression in HOS and MG‐63 osteosarcoma cells (western blotting and real‐time PCR, Figure 2A,B) resulted in diminished cell proliferation (MTT assay, Figure 2C), cell cycle arrest (EdU assay, Figure 2D), increased apoptotic cell ratio (Annexin V staining, Figure 2E), repressed cell colony formation (Figure 2F), retarded cell migration (scratch assay, Figure 2G), and cell invasion (Transwell assay, Figure 2H). Taken together, these findings demonstrate that *DEPDC1* is an oncogene that promotes osteosarcoma cancer cell growth and cell migration while inhibiting cell apoptosis.

### 3.3 Perturbed expression of *DEPDC1* slowing experimental osteosarcoma growth in vivo

**FIGURE 3:**
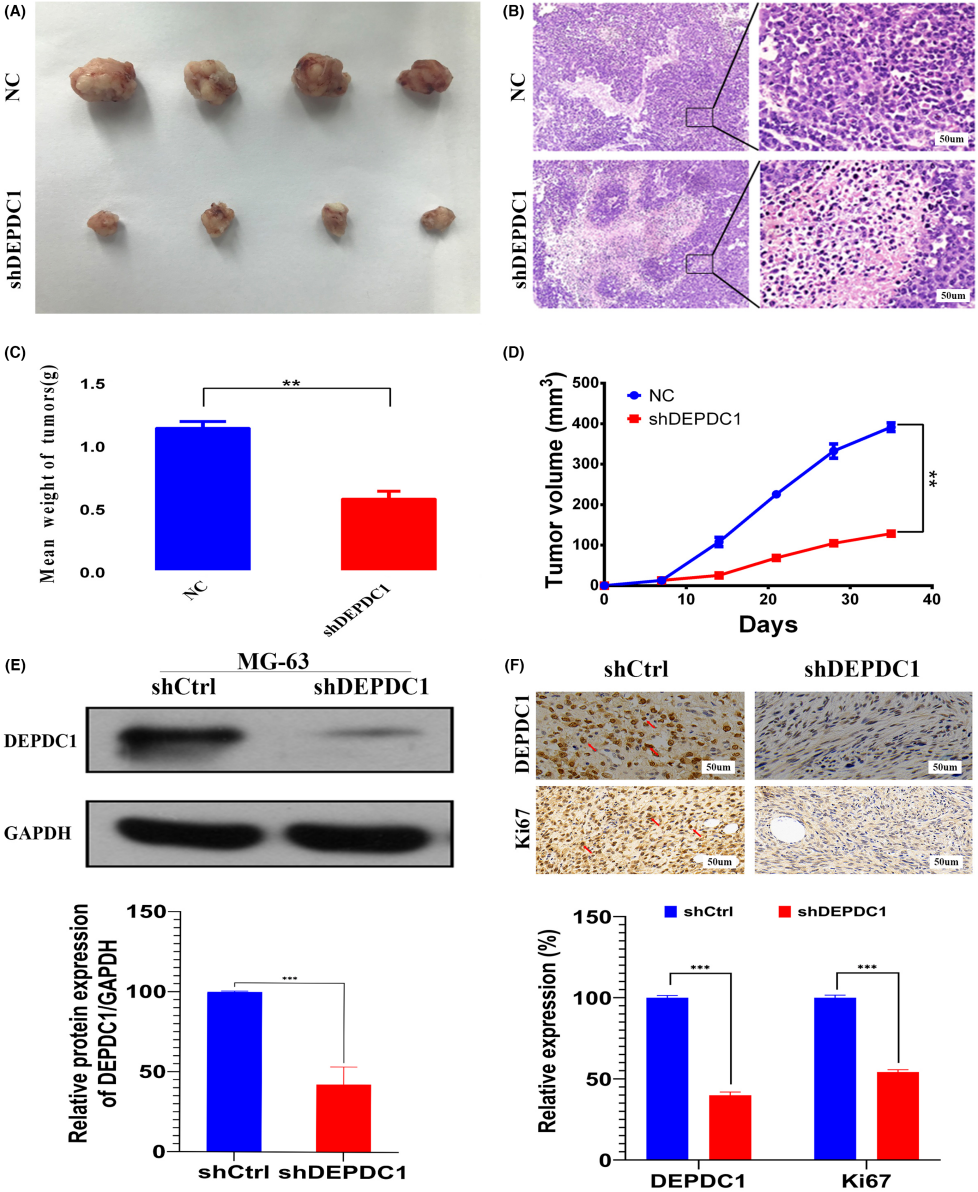
Knockdown of *DEPDC1* decelerating experimental osteosarcoma tumor growth. (A) Macroscopic views of primary tumors retrieved from mouse models inoculated with wild or *DEPDC1*‐knockdown osteosarcoma cells. (B) Representative H&E stained photomicrographs of experimental mouse tumors. (C) Plots of mean tumor weight between groups at sacrifice (**p < 0.01). (D) Summary of tumor volume changes during tumorigenesis after inoculations with wild or sh*DEPDC1*‐treated osteosarcoma cells (**p < 0.01). (E) Western blot of control and sh*DEPDC1* cell lines. (F) IHC against Ki‐67, a proliferation marker.

To investigate the role of *DEPDC1* in the progression of osteosarcoma in vivo, a nude mouse xenograft model was established with subcutaneous inoculation of wild‐type or sh*DEPDC1* transduced osteosarcoma cells. The experimental osteosarcoma tumor sizes from the *DEPDC1*‐knockdown human osteosarcoma cells were dramatically smaller than those inoculated with wild‐type osteosarcoma cells (Figure 3A,C,D). Notably, H&E staining illustrates multi‐focal necrotic areas with cell nuclear condensations and cytoplasmic fibrosis on *DEPDC1*‐knockdown tumor sections (Figure 3B). After silencing the expression of *DEPDC1* (Figure 3E), it was also noticed that Ki‐67, a proliferation marker, was significantly down‐regulated in the osteosarcoma tissue (Figure 3F).

### 3.4 *DEPDC1* expression correlated with human osteosarcoma progression

**FIGURE 4:**
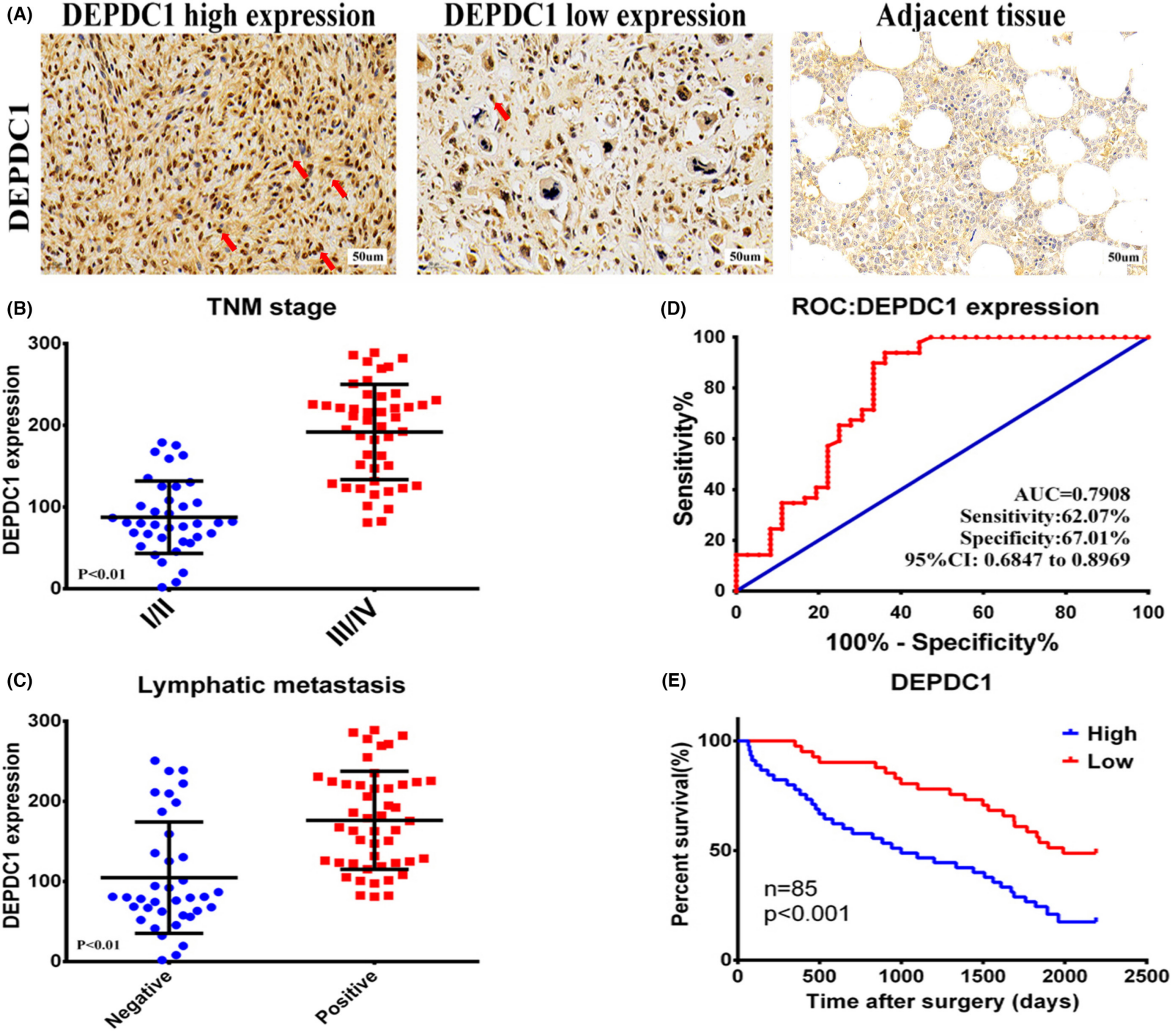
Expression of *DEPDC1* in human osteosarcoma correlated with human osteosarcoma progression. (A) Representative IHC staining image of *DEPDC1* in osteosarcoma and adjacent non‐cancerous tissues. (B) Association between *DEPDC1* expression and TNM stage/lymphatic metastasis in patients with osteosarcoma (*p* < 0.01). (D) Receiver operating characteristic (ROC) curves for predicting patient survival time using *DEPDC1* expression. (E) Kaplan–Meier analysis of overall survival according to *DEPDC1* expression levels (*p* < 0.01). The area under the curve (AUC); tumor node metastasis (TNM).

To explore the relationship between the *DEPDC1* and the clinicopathological characteristics of osteosarcoma patients, we analyzed the positive staining patterns of *DEPDC1* in clinical osteosarcoma specimens (Figure 4A, Table S1) that were correlated with patients' disease progression. The protein expression levels of *DEPDC1* appeared significantly higher in the advanced tumor, node, metastasis (TNM) stage group, and the lymphatic metastasis‐positive group (Figure 4B,C). Indeed, ROC curves indicated that the area under the curve (AUC) of the *DEPDC1*‐based predictions was 0.7908, suggesting that *DEPDC1* could be used to predict the survival rate of osteosarcoma patients (Figure 4D). Importantly, the higher expression levels of *DEPDC1* were greatly associated with the decreased survival time of osteosarcoma patients (*p* < 0.001, Figure 4E).

## 4 DISCUSSION

*DEPDC1* is a newly discovered tumor‐related gene that has a highly conserved domain. Many studies have found that proteins with DEP domains can regulate many cellular functions, such as cell membrane anchoring, signal transduction, cell polarity establishment, and regulation of small molecule guanosine triphosphate (GTP) enzyme activity.^24^ Recent studies have shown that *DEPDC1* is overexpressed in bladder cancer, breast cancer, lung adenocarcinoma, and other malignant tumor types.^9^, ^13^, ^14^ Furthermore, *DEPDC1* may be used in the diagnosis and treatment of various tumors. Kretschmer et al. found that *DEPDC1* and *FOXM1* are significantly upregulated in ductal carcinoma in situ (DCIS) and thus can be used to identify early molecular markers of breast cancer.^25^ The cancer peptide vaccine S‐288310 containing oncoantigens against *DEPDC1* is well tolerated and can effectively prolong the survival time of patients with urothelial carcinoma of the bladder.^11^ However, it remains unclear whether *DEPDC1* is the main mechanism for promoting the proliferation and migration of malignant tumors. In this study, we found that *DEPDC1* is highly expressed in human osteosarcoma through the GEO database analysis and subsequently confirmed its high expression on patient osteosarcoma specimens and commercial osteosarcoma cell lines (Figure 1). Also, we found that *DEPDC1* promotes the proliferation and migration of osteosarcoma in vitro and in vivo (Figures 2, 3). The high expression of *DEPDC1* correlated with the reduced survival time of osteosarcoma patients (Figure 4) indicates that *DEPDC1* is a potential prognostic marker and therapeutic molecular target of osteosarcoma.

For this study, we adopted a subcutaneous osteosarcoma mouse model.^26^, ^27^ Although an in situ mouse experimental proximal bone model has also been broadly used for osteosarcoma research,^28^, ^29^, ^30^, ^31^ the subcutaneous tumor model has the advantage of establishing the direct observation of the tumor growth and being able to readily collect important data such as animal weight, tumor growth curve, and tumor weight. At the same time, a subcutaneous tumor can be used for correlation analysis of tissue and body fluid samples, which is widely used in biomedical research and drug development. The MG‐63 human osteosarcoma cells are a commonly used cell line for in vivo investigation of osteosarcoma,^32^, ^33^ which was used to successfully establish the experimental tumor model in this study. Combined with the outcomes of this animal model study, we are the first to report here that the proliferation and migration of osteosarcoma cells are affected by *DEPDC1* in in vitro and in vivo settings. In addition, high expression of *DEPDC1* promotes the proliferation and migration of multiple malignancies such as breast cancer,^34^ hepatocellular carcinoma,^35^, ^36^ glioma,^37^ and bladder cancer,^38^ and ultimately leads to poor prognosis in patients.^39^

Therefore, we speculate that in human osteosarcoma cells, high expression of *DEPDC1* promotes progression and bad prognosis of human osteosarcoma. The current investigation is underway to explore how *DEPDC1* affects the progression of osteosarcoma and which signaling pathway *DEPDC1* participates in the promotion, proliferation, migration, and apoptosis inhibition of osteosarcoma.

## 5 CONCLUSION

In this study, we determined that *DEPDC1* promotes the proliferation and migration of osteosarcoma cells in vitro and in vivo, which may provide new therapeutic targets for the further development of novel anticancer drugs. At the same time, *DEPDC1* appears to accurately predict the clinical characteristics and prognosis of patients, and may also be critical for osteosarcoma diagnosis and prognosis.

## AUTHOR CONTRIBUTIONS

Lin Shen and Han Li performed experiments, analyzed data, and wrote the manuscript. Chendan Zhou, Ying Zhang, Kai Zhao, Laitong Lu, and Morgan Bretches participated in the experiments and manuscript preparation. Han Li contributed to the animal model. Bin Ning, Ronghan Liu, Shang‐You Yang, and Aijun Zhang conceived the study and participated in manuscript preparation and critical editing.

## FUNDING INFORMATION

This study was supported by the Key Research and Development Program of Shandong Province (2019GSF108060) and the National Science Foundation of Shandong Province (ZR202010220039).

## ETHICS APPROVAL AND CONSENT TO PARTICIPATE

All of these clinical studies were approved by the Institutional Ethical Review Boards of Jinan Central Hospital, and written informed consent was obtained from all patients. All animal experiments were carried out in accordance with the guidelines approved by the Institutional Animal Care and Use Ethics Committee of Shandong University (SYXK20150015).

## CONSENT FOR PUBLICATION

All authors have agreed to publish this manuscript.

## Supporting information


Appendix S1

Table S1

## Data Availability

The datasets used and/or analyzed during the current study are available from the corresponding author upon reasonable request.
