# Supplementary material for: DEPDC1 as a crucial factor in the progression of human osteosarcoma
Source: Cancer Med. 2022 Dec 7;12(5):5798–808. doi: 10.1002/cam4.5340 (PMC10028160; doi:10.1002/cam4.5340)
Supplement: Supplementary file 1 — Appendix S1 [file CAM4-12-5798-s001.pdf]

细胞遗传质量鉴定检验报告

检品名称：细胞系

检验类型：STR 基因型检验

样品编号：

表 1 样本编号

客户样本编号	公司编号
241	20170410-07

样品数量：1

样品性状：细胞系

检测项目：STR

送检单位：上海中乔新舟生物技术有限公司

检测方法：用 Axygen 的基因组抽提试剂盒提取 DNA，采用 20- STR 扩增方案扩增，在 ABI 3730XL 型遗传分析仪上对 STR 位点和性别基因 Amelogenin 进行检测。

检验结果：

(一)检验基本情况

表 2：样本基因型检验结果

	多等位基因	匹配细胞系	细胞库	EV 值	匹配说明
20170410-07	无	HFOB1.19	DSMZ	1	完全匹配

- 多等位基因指三等位及以上基因现象。
- 本次检测各细胞分型结果良好。

(二)各样本描述

(三)20170410-07：该株细胞 DNA 分型在细胞系检索中找到<u>完全匹配</u>的细胞系，DSMZ 数据库显示细胞名为 **hFOB1.19**，细胞号对应 **CRL-11372**。本次检测在该细胞系中**没有发现多等位基因**.

(四)样本分型结果

表 3：细胞 20170410-07 的 STR 位点和 Amelogenin 位点的基因分型结果

Marker	样本				细胞库信息		
	Allele1	Allele2	Allele3	Allele4	Allele1	Allele2	Allele3
D5S818	11	12			11	12	
D13S317	11	12			11	12	
D7S820	8	10			8	10	
D16S539	9	13			9	13	
VWA	16	18			16	18	
TH01	7	9.3			7	9.3	
AMEL	X	X			X	X	
TPOX	11	11			11	11	
CSF1PO	10	13			10	13	
D12S391	20	23					
FGA	19	22					
D2S1338	23	24					
D21S11	29	32.2					
D18S51	10	17					
D8S1179	10	14					
D3S1358	17	18					
D6S1043	13	16					
PENTAE	8	11					
D19S433	13	15					
PENTAD	9	13					

其他说明：

(一)分型方案及位点分布：

附表：实验方案及位点

	方案 1	方案 2	方案 3	方案 4

1	TH01	TPOX	D3S135 8	AMEL
2	D12S39 1	VWA	D13S31 7	D5S818
3	D7S820	D8S1179	D6S104 3	D2S1338
4	CSF1PO	PENTAD	D16S53 9	D21S11
5	FGA		D19S43 3	D18S51
6	PENTAE			

中乔新舟
CELL RESEARCH

Certificate of STR Analysis

Applied Biosystems
GeneMapper 4.0

Cell Line Authentication-12

Sample File	Sample Name	Panel	OS	SQ
55_G07_CellLineAuthentication-0414.fsa	241	STR Profile 1-human		

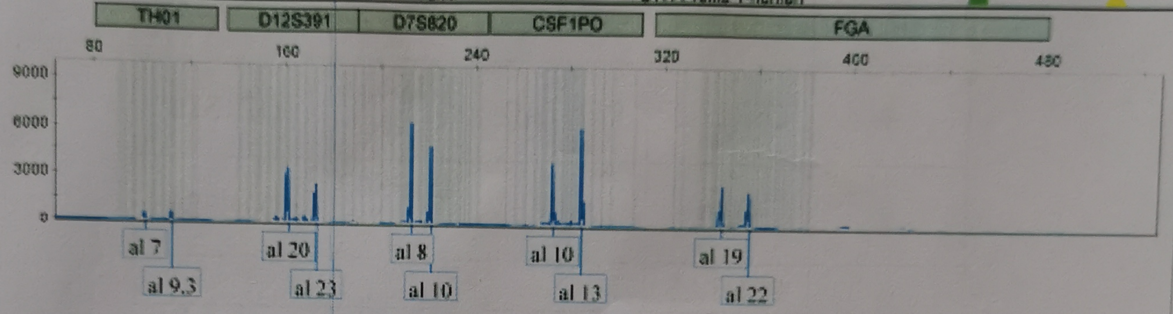

55_G07_CellLineAuthentication-0414.fsa	241	STR Profile 1-human		

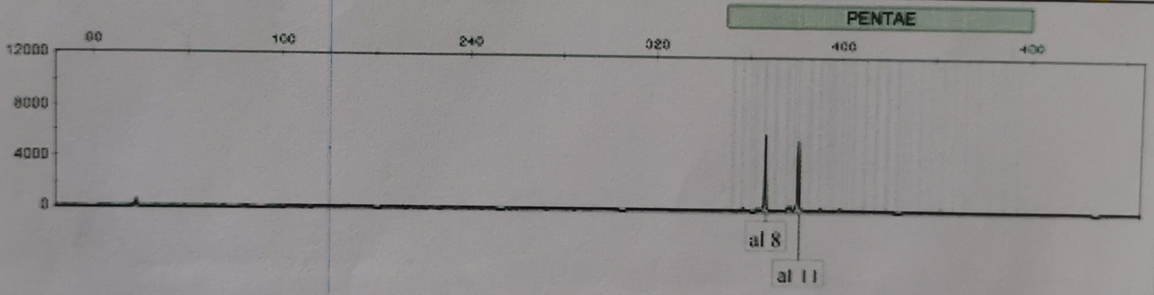

63_G08_GaoYiJing-1-4XiaoShi5-7-0418.fsa	241	STR Profile 2-human		

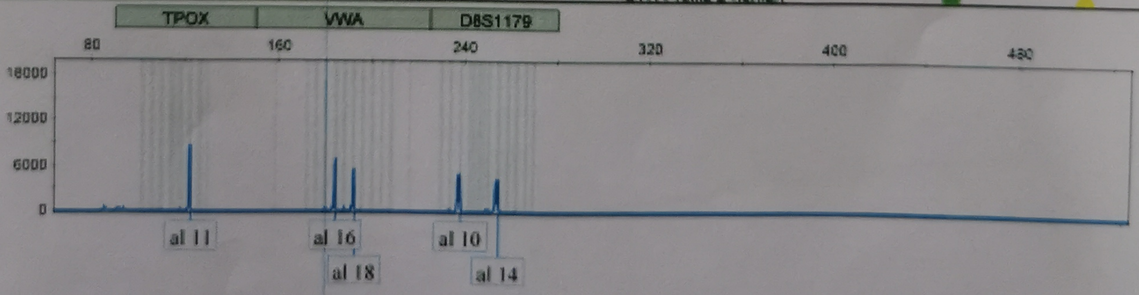

中乔新舟
CELL RESEARCH

Certificate of STR Analysis

Applied Biosystems
GeneMapper 4.0

Cell Line Authentication-12

Sample File	Sample Name	Panel	OS	SQ
63_G05_GaoYiJing_I-4XiaoSh5-7-0418.fsa	241	STR Profile 2-human		

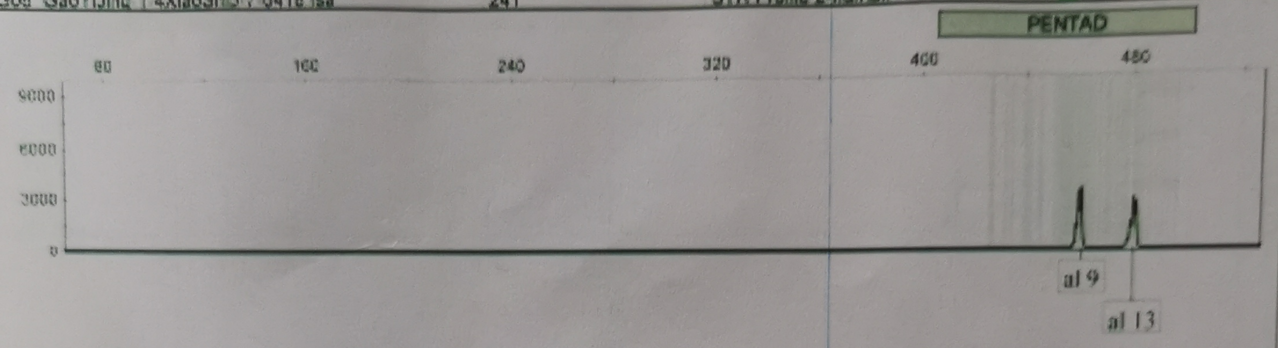

79_G10_MingQingSenMAOA-2(2-9)1-8-0414.fsa	241	STR Profile 3-human		

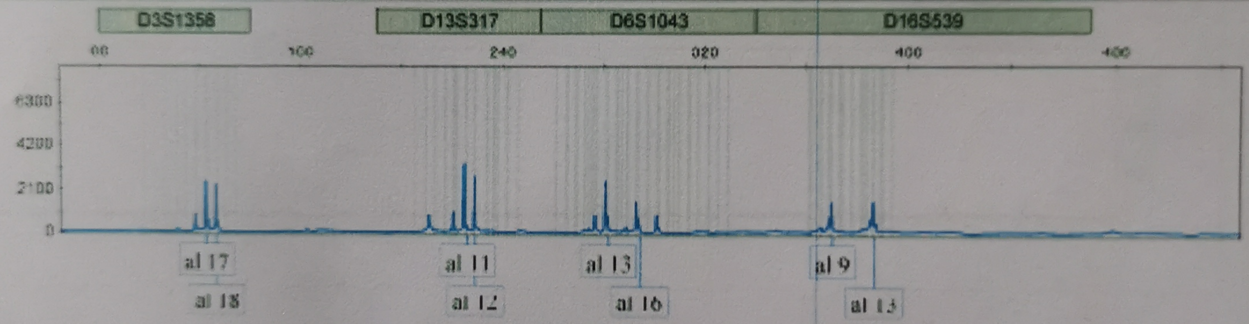

79_G10_MingQingSenMAOA-2(2-9)1-8-0414.fsa	241	STR Profile 3-human		

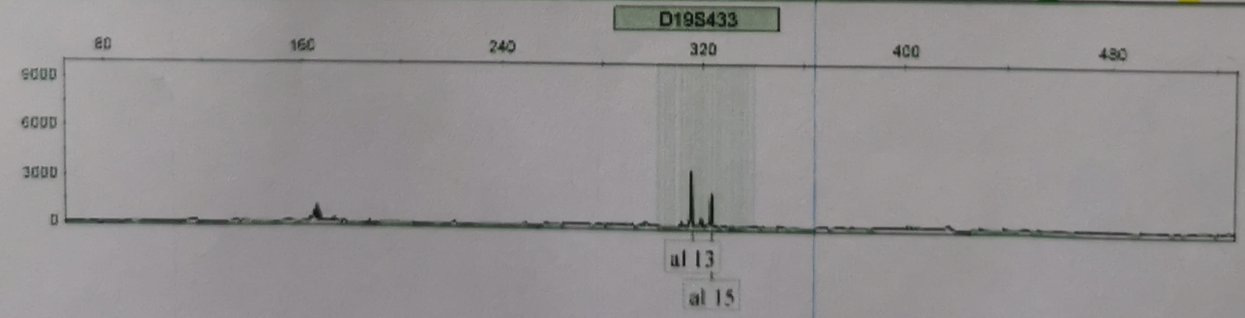

中乔新舟
CELL RESEARCH

Certificate of STR Analysis

Applied Biosystems

Cell Line Authentication-12

GeneMapper 4.0

Sample File	Sample Name	Panel	OS	SQ
15_G02_CellLineAuthentication-0414.fsa	241	STR Profile 4-human		

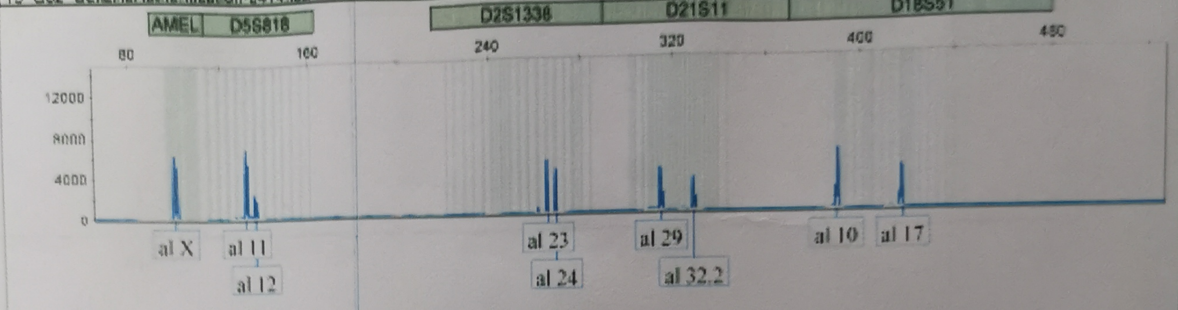

15_G02_CellLineAuthentication-0414.fsa	241	STR Profile 4-human		

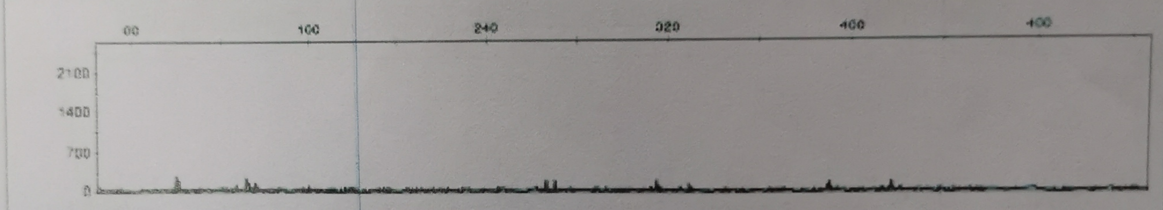

Fri Apr 28.2017 12:17PM. PDT

Printed by: gm

签发日期：
2017 年 05 月

细胞遗传质量鉴定检测

Cell Line Authentication Service

STR 基因型检测报告

送检单位：上海吉凯基因医学科技股份有限公司

检品名称：细胞系

委托单位：上海翼和应用生物技术有限公司

报告日期：2021-07-15

上海翼和应用生物技术有限公司 联系地址：上海市松江区龙腾路 1015 弄中星创意园 2 号 502 室
网址：http://www.biowing.com.cn/邮箱：market@biowing.com.cn 服务热线：021-33559491

报告说明

1. 本报告只对送检的来样负责。
2. 检验报告上的检验结果和检验单位名称，未经同意不得用于广告、评优及商业宣传。
3. 对本报告有异议，请于收到报告之日起十五日内以书面方式提出，逾期不予受理。
4. 对纸质检验报告涂改、增删，或未加盖检验单位印章的复印件均无效。

上海翼和应用生物技术有限公司 联系地址：上海市松江区龙腾路 1015 弄中星创意园 2 号 502 室
网址：http://www.biowing.com.cn/邮箱：market@biowing.com.cn 服务热线：021-33559491

样 品 信 息

样品编号：

客户样本编号	公司编号
HOS	20210713-17

样品数量： 1

样品性状： 细胞系

检测项目： STR

送检单位： **上海吉凯基因医学科技股份有限公司**

检测方法： 用 Axygen 的基因组抽提试剂盒提取 DNA，采用 21- STR 扩增方案扩增，在 ABI 3730XL 型遗传分析仪上对 STR 位点和性别基因 Amelogenin 进行检测。

上海翼和应用生物技术有限公司 联系地址：上海市松江区龙腾路 1015 弄中星创意园 2 号 502 室
网址：http://www.biowing.com.cn/邮箱：market@biowing.com.cn 服务热线：021-33559491

检 测 结 果

(一) 检验基本情况

公司编号	多等位基因	匹配细胞系	细胞库	EV 值	匹配说明
20210713-17	无	HOS	DSMZ	1.0	完全匹配

样本基因型检验结果

- 多等位基因指三等位及以上基因现象。
- 本次检测各细胞分型结果良好。

(二) 各样本描述

- 20210713-17：该株细胞 DNA 分型在细胞系检索中找到**完全匹配**的细胞系，DSMZ 数据库显示细胞名为 **HOS**，细胞号对应 **CRL-1543**。本次检测在该细胞系中**没有发现多等位基因**。

  备注：待测细胞系与收录于 ATCC, DSMZ, JCRB 和 RIKEN 数据库的细胞系 STR 数据进行比对，未收录于以上细胞库的细胞系将无法匹配。

上海翼和应用生物技术有限公司 联系地址：上海市松江区龙腾路 1015 弄中星创意园 2 号 502 室
网址：http://www.biowing.com.cn/邮箱：market@biowing.com.cn 服务热线：021-33559491

（三）样本分型结果

细胞 20210713-17 的 STR 位点和 Amelogenin 位点的基因分型结果						
Loci	送检细胞 STR 信息			细胞库细胞 STR 信息		
	送检细胞名：HOS			细胞库细胞名：HOS		
	Allele1	Allele2	Allele3	Allele1	Allele2	Allele3
D5S818	13	13		13	13	
D13S317	12	12		12	12	
D7S820	11	12		11	12	
D16S539	10	13		10	13	
VWA	18	18		18	18	
TH01	6	6		6	6	
AMEL	X	X		X	X	
TPOX	8	11		8	11	
CSF1PO	12	12		12	12	
D12S391	20	20				
FGA	24	24				
D2S1338	24	25				
D21S11	31.2	32.2				
D18S51	14	14				
D8S1179	14	14				
D3S1358	15	15				
D6S1043	18	18				
PENTAE	7	12				
D19S433	13	13				
PENTAD	9	10				
D1S1656	13	15				

上海翼和应用生物技术有限公司 联系地址：上海市松江区龙腾路 1015 弄中星创意园 2 号 502 室
网址：http://www.biowing.com.cn/邮箱：market@biowing.com.cn 服务热线：021-33559491

其他说明

(一) 分型方案及位点分布

	方案 1	方案 2	方案 3	方案 4
1	D3S1358	D8S1179	D19S433	AMEL
2	VWA	D21S11	TH01	D1S1656
3	D7S820	D16S539	D13S317	D5S818
4	CSF1PO	D2S1338	TPOX	D12S391
5	PENTAE	PENTAD	D18S51	FGA
6			D6S1043	

实验方案及位点

(二) STR 数据库比对

本公司采用 DSMZ tools 进行细胞系比对，其中包含来自于 ATCC, DSMZ, JCRB 和 RIKEN 数据库的 2455 个细胞系 STR 数据。如果待检测细胞未收录于以上细胞库或这是自行建立的新细胞系将无法进行比对，用户需根据细胞分型结果自行与其他数据库进行比对。

(三) 文献引用参考

1．Authentication testing of HEK 293T and HeLa cell lines have been performed by Shanghai Biowing Applied Biotechnology Co.,Ltd via STR profiling. STR profiles match the standards rec ommended for HEK 293T and HeLa cell lines authentication

2．AGS, NCI-N87, HGC-27 and HEK293 were STR-authenticated on Dec. 8, 2015 by Shanghai Biowing Applied Biotechnology Co. LTD, Shanghai, China

主要实验人员：张佳男

复核人: 钱宁

负责人： 白杨

签发日期：2021-07-15

上海翼和应用生物技术有限公司 联系地址：上海市松江区龙腾路 1015 弄中星创意园 2 号 502 室
网址：http://www.biowing.com.cn/邮箱：market@biowing.com.cn 服务热线：021-33559491

细胞遗传质量鉴定检测

Cell Line Authentication Service

STR 基因型检测报告

送检单位：上海吉凯基因医学科技股份有限公司

检品名称：细胞系

委托单位：上海翼和应用生物技术有限公司

报告日期：2021-06-02

上海翼和应用生物技术有限公司 联系地址：上海市松江区龙腾路 1015 弄中星创意园 2 号 502 室
网址：http://www.biowing.com.cn/邮箱：market@biowing.com.cn 服务热线：021-33559491

报 告 说 明

1. 本报告只对送检的来样负责。
2. 检验报告上的检验结果和检验单位名称，未经同意不得用于广告、评优及商业宣传。
3. 对本报告有异议，请于收到报告之日起十五日内以书面方式提出，逾期不予受理。
4. 对纸质检验报告涂改、增删，或未加盖检验单位印章的复印件均无效。

上海翼和应用生物技术有限公司 联系地址：上海市松江区龙腾路 1015 弄中星创意园 2 号 502 室
网址：http://www.biowing.com.cn/邮箱：market@biowing.com.cn 服务热线：021-33559491

样 品 信 息

样品编号：

客户样本编号	公司编号
MG-63	20210531-09

样品数量： 1

样品性状： 细胞系

检测项目： STR

送检单位： **上海吉凯基因医学科技股份有限公司**

检测方法： 用 Axygen 的基因组抽提试剂盒提取 DNA，采用 21- STR 扩增方案扩增，在 ABI 3730XL 型遗传分析仪上对 STR 位点和性别基因 Amelogenin 进行检测。

上海翼和应用生物技术有限公司　联系地址：上海市松江区龙腾路 1015 弄中星创意园 2 号 502 室
网址：http://www.biowing.com.cn/邮箱：market@biowing.com.cn　服务热线：021-33559491

检 测 结 果

(一) 检验基本情况

公司编号	多等位基因	匹配细胞系	细胞库	EV 值	匹配说明
20210531-09	无	MG-63	EXPASY	0.98	基本匹配

样本基因型检验结果

- 多等位基因指三等位及以上基因现象。
- 本次检测各细胞分型结果良好。

(二) 各样本描述

- 20210531-09：该株细胞 DNA 分型在细胞系检索中找到**基本匹配**的细胞系，EXPASY 数据库显示细胞名为 **MG-63**，细胞号对应 **CVCL_0426 Best**。本次检测在该细胞系中**没有发现多等位基因**。

备注：待测细胞系与收录于 ATCC, DSMZ, JCRB 和 RIKEN 数据库的细胞系 STR 数据进行比对，未收录于以上细胞库的细胞系将无法匹配。

上海翼和应用生物技术有限公司 联系地址：上海市松江区龙腾路 1015 弄中星创意园 2 号 502 室
网址：http://www.biowing.com.cn/邮箱：market@biowing.com.cn 服务热线：021-33559491

（三）样本分型结果

细胞 20210531-09 的 STR 位点和 Amelogenin 位点的基因分型结果						
Loci	送检细胞 STR 信息			细胞库细胞 STR 信息		
	送检细胞名：MG-63			细胞库细胞名：MG-63		
	Allele1	Allele2	Allele3	Allele1	Allele2	Allele3
D5S818	11	12		11	12	
D13S317	11	11		11	11	
D7S820	10	10		10	10	
D16S539	11	11		11	11	
VWA	16	19		16	19	
TH01	9.3	9.3		9.3	9.3	
AMEL	X	Y		X	Y	
TPOX	8	11		8	11	
CSF1PO	10	12		10	12	
D12S391	15	20				
FGA	21	25		21	25	
D2S1338	17	24		17	24	
D21S11	30	30		30	30	
D18S51	12	16		16	16	
D8S1179	13	13		13	13	
D3S1358	15	15		15	15	
D6S1043	12	17				
PENTAE	11	12		11	12	
D19S433	13	14		13	14	
PENTAD	9	13		9	13	
D1S1656	14	16				

上海翼和应用生物技术有限公司 联系地址：上海市松江区龙腾路 1015 弄中星创意园 2 号 502 室
网址：http://www.biowing.com.cn/邮箱：market@biowing.com.cn 服务热线：021-33559491

其他说明

(一) 分型方案及位点分布

	方案 1	方案 2	方案 3	方案 4
1	D3S1358	D8S1179	D19S433	AMEL
2	VWA	D21S11	TH01	D1S1656
3	D7S820	D16S539	D13S317	D5S818
4	CSF1PO	D2S1338	TPOX	D12S391
5	PENTAE	PENTAD	D18S51	FGA
6			D6S1043	

实验方案及位点

(二) STR 数据库比对

本公司采用 DSMZ tools 进行细胞系比对，其中包含来自于 ATCC, DSMZ, JCRB 和 RIKEN 数据库的 2455 个细胞系 STR 数据。如果待检测细胞未收录于以上细胞库或这是自行建立的新细胞系将无法进行比对，用户需根据细胞分型结果自行与其他数据库进行比对。

(三) 文献引用参考

1．Authentication testing of HEK 293T and HeLa cell lines have been performed by Shanghai Biowing Applied Biotechnology Co.,Ltd via STR profiling. STR profiles match the standards rec ommended for HEK 293T and HeLa cell lines authentication

2．AGS, NCI-N87, HGC-27 and HEK293 were STR-authenticated on Dec. 8, 2015 by Shanghai Biowing Applied Biotechnology Co. LTD, Shanghai, China

主要实验人员：张佳男

复核人: 钱宁

负责人： 白杨

签发日期：2021-06-02

Cell Line Saos-2 STR Profile Report

Cell Line Designation: Saos-2

Date Sample Received: Oct 20th, 2017

Report Date: Oct 31th, 2017

Methodology: Twenty short tandem repeat (STR) loci plus the gender determining locus,Amelogenin, were amplified using the commercially available PowerPlex® 21 System from Promega Corporation. The amplified products were processed using the Applied Biosystems 3730xl DNA Analyzer. Data were analyzed using GeneMapper 5.0 software (Applied Biosystems). Appropriate positive and negative controls were run and confirmed for each sample submitted.

Data Interpretation: Cell lines were authenticated using Short Tandem Repeat (STR) analysis as described in 2012 in ANSI Standard (ASN-0002) by the ATCC Standards Development Organization (SDO) and in Capes-Davis et al., Match criteria for human cell line authentication: Where do we draw the line? Int J Cancer.2013;132(11):2510-9.

Genechem

GENECHEM performs STR Profiling following ISO 9001:2008 and ISO/IEC 17025:2005 quality standards.

There are no warranties with respect to the services or results supplied, express or implied, including, without limitation, any implied warranty of merchantability or fitness for a particular purpose. GENECHEM is not liable for any damages or injuries resulting from receipt and/or improper, inappropriate, negligent or other wrongful use of the test results supplied, and/or from misidentification, misrepresentation, or lack of accuracy of those results. Your exclusive remedy against GENECHEM and those supplying materials used in the services for any losses or damage of any kind whatsoever, whether in contract, tort, or otherwise, shall be, at GENECHEM 's option, refund of the fee paid for such service or repeat of the service.

TEST RESULTS:

Test Results for Submitted Sample		Reference Database Profile
**Loci**	**Query Profile: Saos-2**	**Database Profile: Saos-2**
**Amelogenin**	X	X
**D13S317**	12 13	12 13
**D16S539**	12 13	12 13
**CSF1PO**	10	10
**TH01**	6 9	6 9
**vWA**	18	18
**D7S820**	8 10	8 10
**D5S818**	12	12
**TPOX**	8	8
**D3S1358**	14 18	
**D1S1656**	15	
**D6S1043**	11 14	
**Penta E**	14 19	
**D18S51**	15	
**D2S1338**	18	
**Penta D**	11 12	
**D8S1179**	10 12	
**D12S391**	20	
**D19S433**	13	
**FGA**	22 25	
**D21S11**	28 30	
Note: The top nine Loci (8 core STR loci plus Amelogenin) can be made public to verify cell identity. In order to protect the identity of the donor, ***please do not publish*** *the allele calls from all the STR loci tested.*		

Genechem

Percent Match: **100%**

The submitted profile is an exact match for the following human cell line(s) in the reference database (8 core loci plus Amelogenin): **Saos-2**

Explanation of Test Results

Cell lines with ≥80% match are considered to be related; i.e., derived from a common ancestry. Cell lines with between a 55% to 80% match require further profiling for authentication of relatedness. Reference Database Profile was obtained from ExPASy, DSMZ, ATCC or China National Infrastructure of Cell Line Resource STR database.

Applied Biosystems
GeneMapper Software 5

0810-final

Sample File	Sample Name	Panel	OS	SQ
106_H05.fsa	H05	PowerPlex_21_POP7_Panels_v2.0		

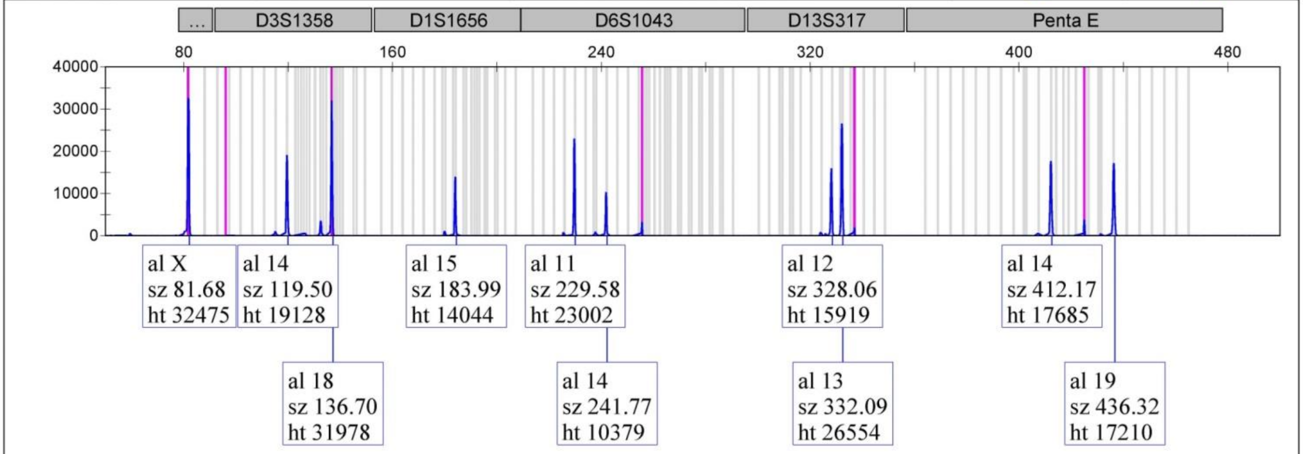

106_H05.fsa	H05	PowerPlex_21_POP7_Panels_v2.0		

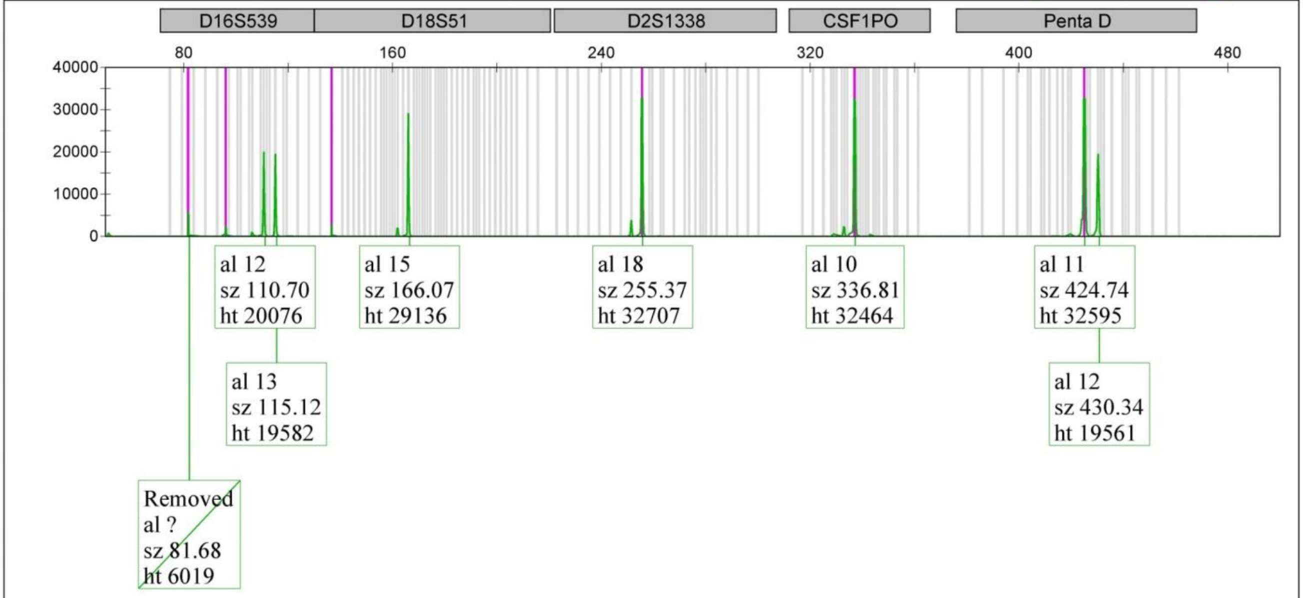

Tue Oct 24,2017 03:30PM, CST   Printed by: gm   Page 1 of 2

Applied Biosystems
GeneMapper Software 5

0810-final

Sample File	Sample Name	Panel	OS	SQ
106_H05.fsa	H05	PowerPlex_21_POP7_Panels_v2.0		

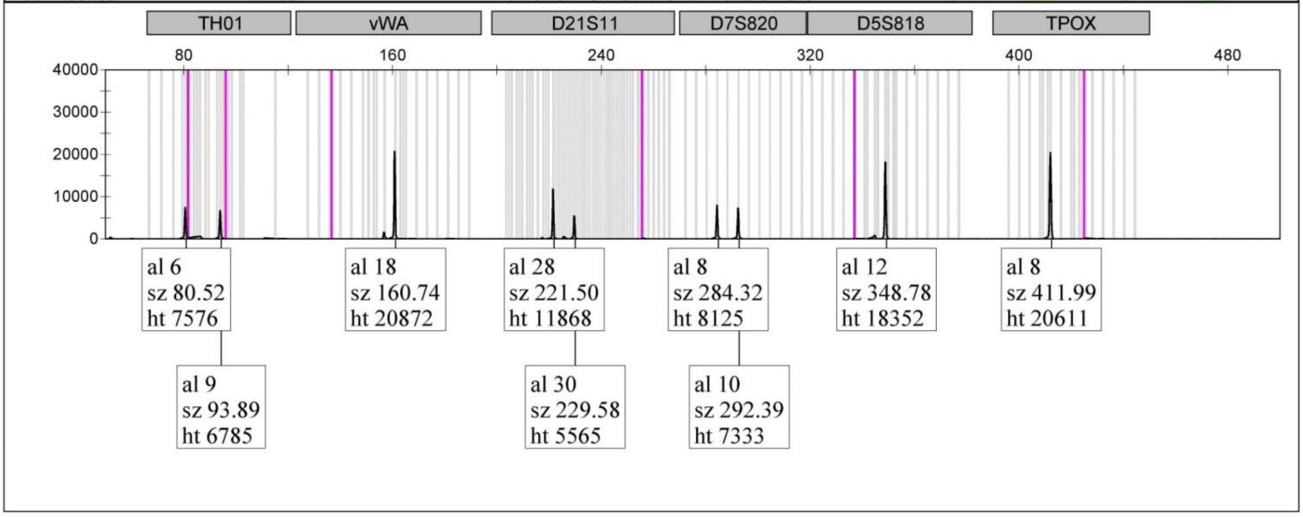

106_H05.fsa	H05	PowerPlex_21_POP7_Panels_v2.0		

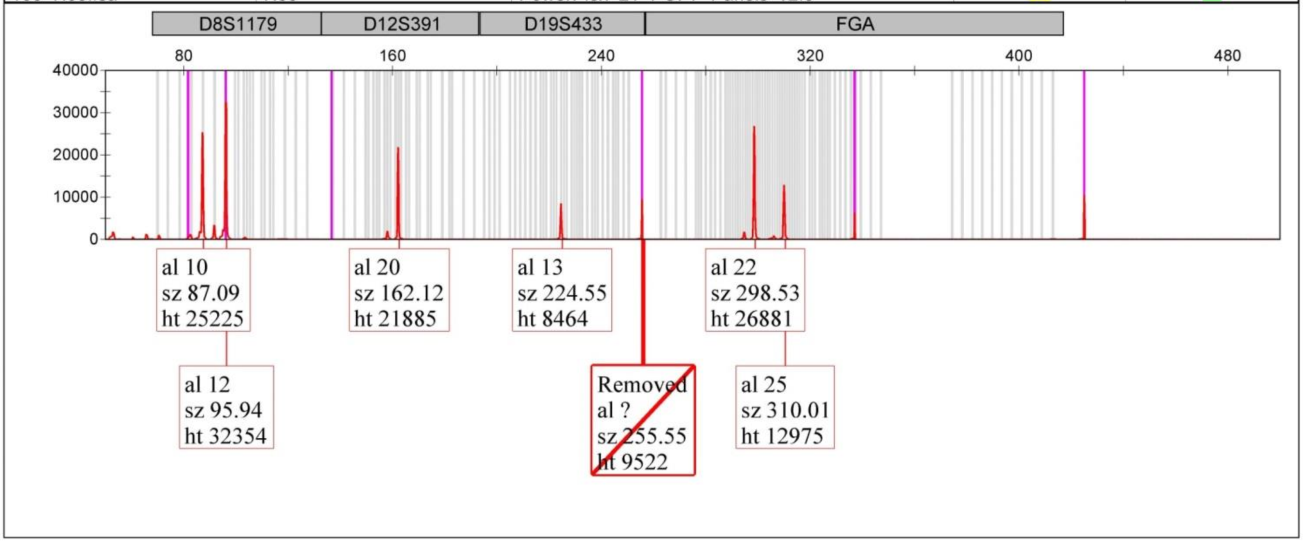

Tue Oct 24,2017 03:30PM, CST Printed by: gm Page 2 of 2

Shanghai Genechem Co., Ltd.
Add：NO.332, Aidisheng Road, Zhangjiang Hi-Tech Park, Shanghai 201203 P.R. China Tel : (86) 21 51320189
Fax : (86) 21 51370635

细 胞 遗 传 质 量 鉴 定 检 测

Cell Line Authentication Service

STR 基 因 型 检 测 报 告

送检单位：上海吉凯基因医学科技股份有限公司

检品名称：细胞系

委托单位：上海翼和应用生物技术有限公司

报告日期：2021-03-29

上海翼和应用生物技术有限公司联系地址：上海市松江区龙腾路 1015 弄中星创意园 2 号 502 室
网址：http://www.biowing.com.cn/邮箱：market@biowing.com.cn　　服务热线：021-33559491

报 告 说 明

1. 本报告只对送检的来样负责。
2. 检验报告上的检验结果和检验单位名称，未经同意不得用于广告、评优及商业宣传。
3. 对本报告有异议，请于收到报告之日起十五日内以书面方式提出，逾期不予受理。
4. 对纸质检验报告涂改、增删，或未加盖检验单位印章的复印件均无效。

上海翼和应用生物技术有限公司联系地址：上海市松江区龙腾路 1015 弄中星创意园 2 号 502 室
网址：http://www.biowing.com.cn/邮箱：market@biowing.com.cn 服务热线：021-33559491

样 品 信 息

样品编号：

客户样本编号	公司编号
U-20S	20210322-11

样品数量： 1

样品性状： 细胞系

检测项目： STR

送检单位： **上海吉凯基因医学科技股份有限公司**

检测方法： 用 Axygen 的基因组抽提试剂盒提取 DNA，采用 21- STR 扩增方案扩增，在 ABI 3730XL 型遗传分析仪上对 STR 位点和性别基因 Amelogenin 进行检测。

检测结果

(一) 检验基本情况

公司编号	多等位基因	匹配细胞系	细胞库	EV 值	匹配说明
20210322-11	无	U-2 OS	DSMZ	1.0	完全匹配

样本基因型检验结果

- 多等位基因指三等位及以上基因现象。
- 本次检测各细胞分型结果良好。

(二) 各样本描述

- 20210322-11：该株细胞 DNA 分型在细胞系检索中找到**完全匹配**的细胞系，DSMZ 数据库显示细胞名为 **U-2 OS**，细胞号对应 **HTB-96**。本次检测在该细胞系中**没有发现多等位基因**。

  备注：待测细胞系与收录于 ATCC, DSMZ, JCRB 和 RIKEN 数据库的细胞系 STR 数据进行比对，未收录于以上细胞库的细胞系将无法匹配。

上海翼和应用生物技术有限公司联系地址：上海市松江区龙腾路 1015 弄中星创意园 2 号 502 室
网址：http://www.biowing.com.cn/邮箱：market@biowing.com.cn 服务热线：021-33559491

（三）样本分型结果

细胞 20210322-11 的 STR 位点和 Amelogenin 位点的基因分型结果						
Loci	送检细胞 STR 信息			细胞库细胞 STR 信息		
	送检细胞名：U-2OS			细胞库细胞名：U-2 OS		
	Allele1	Allele2	Allele3	Allele1	Allele2	Allele3
D5S818	11	11		11	11	
D13S317	13	13		13	13	
D7S820	11	12		11	12	
D16S539	11	12		11	12	
VWA	14	18		14	18	
TH01	6	9.3		6	9.3	
AMEL	X	X		X	X	
TPOX	11	12		11	12	
CSF1PO	13	13		13	13	
D12S391	19	20				
FGA	20	20				
D2S1338	20	24				
D21S11	31	31				
D18S51	12	14				
D8S1179	12	14				
D3S1358	16	16				
D6S1043	11	11				
PENTAE	10	13				
D19S433	15	15				
PENTAD	9	9				
D1S1656	16	17.3				

其 他 说 明

(一) 分型方案及位点分布

	方案 1	方案 2	方案 3	方案 4
1	D3S1358	D8S1179	D19S433	AMEL
2	VWA	D21S11	TH01	D1S1656
3	D7S820	D16S539	D13S317	D5S818
4	CSF1PO	D2S1338	TPOX	D12S391
5	PENTAE	PENTAD	D18S51	FGA
6			D6S1043	

实验方案及位点

(二) STR 数据库比对

本公司采用 DSMZ tools 进行细胞系比对，其中包含来自于 ATCC, DSMZ, JCRB 和 RIKEN 数据库的 2455 个细胞系 STR 数据。如果待检测细胞未收录于以上细胞库或这是自行建立的新细胞系将无法进行比对，用户需根据细胞分型结果自行与其他数据库进行比对。

(三) 文献引用参考

1．Authentication testing of HEK 293T and HeLa cell lines have been performed by Shanghai Biowing Applied Biotechnology Co.,Ltd via STR profiling. STR profiles match the standards rec ommended for HEK 293T and HeLa cell lines authentication

2．AGS, NCI-N87, HGC-27 and HEK293 were STR-authenticated on Dec. 8, 2015 by Shanghai Biowing Applied Biotechnology Co. LTD, Shanghai, China

主要实验人员：张佳男

复核人: 钱宁

负责人：白杨

签发日期：2021-03-29

上海翼和应用生物技术有限公司联系地址：上海市松江区龙腾路 1015 弄中星创意园 2 号 502 室
网址：http://www.biowing.com.cn/邮箱：market@biowing.com.cn 服务热线：021-33559491
